# Evaluation of P22 Antigenic Complex for the Immuno-Diagnosis of Tuberculosis in BCG Vaccinated and Unvaccinated Goats

**DOI:** 10.3389/fvets.2020.00374

**Published:** 2020-07-03

**Authors:** Claudia Arrieta-Villegas, José Antonio Infantes-Lorenzo, Javier Bezos, Miriam Grasa, Enric Vidal, Irene Mercader, Mahavir Singh, Mariano Domingo, Lucía de Juan, Bernat Pérez de Val

**Affiliations:** ^1^IRTA, Centre de Recerca en Sanitat Animal (CReSA, IRTA-UAB), Campus Universitat Autònoma de Barcelona, Barcelona, Spain; ^2^Servicio de Inmunología Microbiana, Centro Nacional de Microbiología, Instituto de Investigación Carlos III, Madrid, Spain; ^3^VISAVET Health Surveillance Center, Universidad Complutense de Madrid, Madrid, Spain; ^4^Departamento de Sanidad Animal, Universidad Complutense de Madrid, Madrid, Spain; ^5^Agrupació de Defensa Sanitària de Cabrum i Oví Lleter de Catalunya, Barbens, Spain; ^6^Departament d'Agricultura, Ramaderia, Pesca i Alimentació de la Generalitat de Catalunya, Barcelona, Spain; ^7^Lionex Diagnostics and Therapeutics GmbH, Braunschweig, Germany; ^8^Departament de Sanitat i Anatomia Animals, Universitat Autònoma de Barcelona (UAB), Barcelona, Spain

**Keywords:** tuberculosis, diagnosis, goats, bacille Calmette-Guérin (BCG), skin test, interferon-gamma release assay (IGRA), serology, P22

## Abstract

Current eradication strategies of tuberculosis (TB) in goats mainly rely on the single intradermal tuberculin test (SIT) and single intradermal cervical comparative tuberculin tests (SICCTs). TB vaccination has been proposed as a cost-effective option in high-prevalence herds or countries where economic compensation for the slaughter of positive animals is not affordable. However, TB vaccination compromises the efficiency of tuberculin-based diagnostic tests. In this study, the performance of a new diagnostic platform, based on the P22 antigenic complex, was assessed for skin test (ST), interferon-gamma release assay (IGRA), and serology under different TB scenarios. The sensitivity (Se) of diagnostic tests was assessed in TB-infected goats from the same farm (herd A, *N* = 77). The specificity (Sp) was assessed in two TB-negative farms (both vaccinated against paratuberculosis): one TB unvaccinated (herd B, *N* = 77) and another vaccinated with bacille Calmette-Guérin (BCG) (herd C, *N* = 68). The single (s) P22-IGRA showed the highest Se among IGRA tests (91%), and the comparative (c) P22-ST showed the highest Sp (100% in herd B and 98% in herd C). Combined interpretation of techniques enabled the best diagnostic performances. Combining the SICCT + sP22-IGRA improved Se (97%) compared to SICCT + tuberculin-based IGRA (95%), with a reduction of Sp (95 and 100%, respectively). Besides, combination of P22-ELISA with cP22-ST or SICCT elicited a similar performance in the non-vaccination context (Se: 94 and 95%; Sp: 95 and 95%, respectively), but Sp was significantly higher for the combination with cP22-ST compared to SICCT in the TB vaccination context (95 and 79%, respectively). The combination of serological tests based on P22 and MPB83 showed higher complementarity and improved 13 percentage points the Se of P22-ELISA alone. These findings suggest that either cell-mediated or antibody-based diagnostic techniques, using the P22 antigen complex, can contribute to improve the immunodiagnostics of TB in goats under different TB control strategies.

## Introduction

Tuberculosis (TB) in goats is a chronic infectious disease, mainly caused by *Mycobacterium bovis* and *Mycobacterium caprae*, members of the *Mycobacterium tuberculosis* complex (MTBC). This disease entails important economic costs for livestock industries (1) and could be a source of TB for cattle (2), other domestic animals (3, 4), wildlife (5), and humans (6).

Spain has the second-highest goat census of the EU, with 2.7 million goat heads (data extracted from FAOSTAT on 17/02/2020). Besides, the high TB burden in goats could explain a number of new bovine TB breakdowns, hampering the goal of TB eradication in cattle (7). Therefore, some regions with a high concentration of caprine herds carry out TB eradication campaigns in caprine flocks (8); however, goat herds are still not subjected to a national eradication program, except for those epidemiologically linked with cattle (9).

The cornerstone of an efficient caprine TB eradication program is the diagnosis. The Spanish bovine TB eradication program effectiveness is highly dependent on the routine tuberculin skin testing (10). Current bovine TB testing is based on the single intradermal tuberculin test (SIT) and single intradermal cervical comparative tuberculin tests (SICCTs), and the interferon-gamma release assay (IGRA). However, in goats under certain epidemiological contexts, those diagnostic tests have some drawbacks in terms of sensitivity (Se) and specificity (Sp) (8, 11).

Another concern for TB diagnostics is the vaccination against *Mycobacterium avium* subsp. *paratuberculosis* (MAP), which has been largely implemented in small ruminants, to prevent the development of clinical disease (12). Nevertheless, even though MAP vaccines are authorized (e.g., Gudair^®^ vaccine), it has been demonstrated that paratuberculosis (PTB) vaccination interferes with STs and IGRA used for TB diagnosis (13, 14). In addition, the efficacy of *M. bovis* bacille Calmette-Guérin (BCG) vaccine has also been assessed in goats during the last decade in different vaccination trials (15–19). Even though these trials showed that BCG conferred certain protection to experimentally and naturally infected goats, it was evidenced that vaccination interfered with current TB diagnostic tests (16, 20).

To overcome diagnostic interferences due to BCG vaccination, defined antigens to differentiate infected from vaccinated animals (DIVA) have been developed (14, 21); nevertheless, those antigens have shown lower Se compared to tests based on standard tuberculins (22). Recently, a new multi-protein complex called P22, obtained from purified protein derivative of *M. bovis* (PPD-B) by affinity chromatography, has been developed (23), yielding high Se and variable Sp, depending on the animal species and epidemiological contexts (24). To date, this antigen has been tested to detect humoral response against MTBC in different species (25–30); however, there is a lack of information regarding its performance for cell-mediated immunity (CMI)-based diagnostics.

The aim of this study was to evaluate the performance of different cell-mediated and humoral immunodiagnostic tests, based on the P22 antigenic complex, for the diagnosis of TB in goats under different epidemiological and control scenarios.

## Materials and Methods

### Herds and Experimental Design

A total of 222 goats from three herds were included in the study (Table 1): 77 infected goats (infection was confirmed postmortem by gross lesions, histopathology or mycobacterial culture, or both) from a TB-positive herd of murciana-granadina goats (herd A); 77 goats belonging to an officially TB-free herd of alpine goats (herd B) that were vaccinated against PTB with Gudair (CZ Vaccines, Porriño, Spain), around 2 years before sampling; and 68 goats from another TB-free herd (herd C) of Blanca de Rasquera autochthonous breed, that were vaccinated against PTB (Gudair^®^) and against TB with *M. bovis* BCG Danish 1,331 strain (ATCC, Ref. 35733) as described previously (15). In herd C, 50% of goats were vaccinated with BCG and Gudair^®^ 9–10 months before sampling, and the remaining 50% were vaccinated more than 1 year before. STs, IGRAs, and immunoglobulin G (IgG) enzyme-linked immunosorbent assays (ELISAs) were carried out in the 77 infected goats, as well as in 138, 142, and 142 noninfected goats, respectively (Table 1).

**Table 1:** Herd and treatment distribution of tested animals.

				**No. of animals tested**		
**Herd**	**TB status**	**BCG^1^**	**Gudair^^®^^^2^**	**ST**	**IGRA**	**ELISA**
A	Positive	No	No	77	77	77
B	Free	No	Yes	77	74	74
C	Free	Yes	Yes	61	68	68

1 *BCG, bacilli Calmette-Guérin Mycobacterium bovis vaccine*.

2 *Gudair^^®^^ vaccine, vaccine against paratuberculosis (Mycobacteium avium subspecies paratuberculosis)*.

*TB, tuberculosis; ST, skin test; IGRA, interferon-gamma release assay*.

Two TB control scenarios were hypothesized in order to study the performance of each diagnostic test: the conventional (TB unvaccinated) scenario, using data from herds A and B, and the BCG-vaccinated (TB-VAC) scenario, using data from herds A and C. Se was calculated using data from herd A, and Sp was calculated using data from herds B and C depending on TB control scenario (Table 2).

**Table 2:** TB control scenarios distribution of tested animals.

				**No. of animals tested**		
**Control scenario**	**Herds**	**BCG^1^**	**Gudair^^®^^^2^**	**ST**	**IGRA**	**ELISA**
Conventional^a^	A+B	No	Yes	154	151	151
TB-VAC^b^	A+C	Yes	Yes	138	145	145

a *Conventional scenario: composed by TB unvaccinated goats*.

b *TB-VAC Scenario: TB negative animals from herd C were vaccinated with BCG and TB-positive animals from herd A were not vaccinated*.

1 *BCG, bacilli Calmette-Guérin Mycobacterium bovis vaccine*.

2 *Gudair^^®^^ vaccine, vaccine against paratuberculosis (Mycobacteium avium subspecies paratuberculosis)*.

*TB, tuberculosis; ST, skin test; IGRA, interferon-gamma release assay*.

### Antigens

*M. tuberculosis* var. *bovis* (PPD-B) and *M. avium* (PPD-A) tuberculins (2,500 IU/ml) were obtained from CZ Vaccines and used at concentrations recommended by the Spanish Ministry (9). The protein complex P22 was produced by immunopurification of PPD-B (CZ Vaccines) as described previously (23) and prepared at a concentration of 500 μg/ml (unpublished data). The DIVA reagent based on a cocktail of recombinant ESAT-6 and CFP-10 proteins (500 μg/ml) (31) and the recombinant MPB83 (MPT83) protein (500 μg/ml) (32) were purchased from Lionex (Braunschweig, Germany).

### Skin Tests

SIT was performed by intradermal inoculation of 0.1 ml of PPD-B in the left-hand side of the neck by using a Dermojet^®^ syringe (Akra Dermojet, Pau, France). In the same way, SICCT was performed by intradermal inoculation of 0.1 ml of PPD-B and PPD-A, both in the left-hand side of the neck, at the proximal and distal parts of the neck, respectively. Besides, 0.1 ml of P22 (at 500 μg/ml) was inoculated in the right-hand side of the neck. The increase in skinfold thickness (SFT) was measured just before the inoculation and after 72 h. Severe interpretations of SIT and SICCT were performed, as previously described in the manual of the Spanish bovine TB eradication program (9). Briefly, positive criterion for SIT: SFT PPD-B > 2 mm (severe); and for SICCT: positive to SIT and SFT PPD-B - SFT PPD-A > 1 mm (severe) or presence of clinical signs in the PPD-B inoculation site. P22 single and comparative STs (sP22-ST and cP22-ST) were interpreted using the same criteria as SIT and SICCT, respectively, i.e., considering SFT P22 and SFT P22 - SFT PPD-A measures, respectively.

### Whole-Blood Interferon-Gamma Release Assays

Blood samples were collected from the jugular vein prior to ST performance using heparinized tubes and were processed as described previously (16). Shortly, blood samples were stimulated with PPD-B, PPD-A, and P22 at a final concentration of 20 μg/ml, and with DIVA reagent (ESAT-6/CFP-10) at 20 μg/ml, while PBS was added as an unstimulated control. Samples were incubated at 37 ± 1°C with 0.5% CO_2_ overnight. Finally, plasma supernatant was collected and analyzed by ELISA (BOVIGAM^®^, Thermo Fisher Scientific, Waltham, MA, USA) and read at 450 nm using a spectrophotometer (Biotek Power Wave XS). The interpretation of tuberculin-based IGRA (STAND-IGRA) results was performed according to the cutoff point recommended by the manufacturer, i.e., the criterion for positivity: PPD-B OD – PBS OD ≥ 0.05 and PPD-B OD > PPD-A OD. Similarly, cP22-IGRA was considered positive when P22 OD – PBS OD ≥ 0.05 and P22 OD > PPD-A OD, whereas sP22-IGRA and DIVA-IGRA were considered positive when P22 OD – PBS OD ≥ 0.05 and DIVA OD – PBS OD ≥ 0.05, respectively.

### Antibody Detection Tests

Plasma samples were analyzed for antibody detection by using two in-house indirect ELISA, one for detecting MPB83 antigen, performed and interpreted as described previously (33), and another one for detecting P22, performed as described previously (24, 25). P22-ELISA was interpreted as follows: ELISA percentage (E%) = [mean sample OD/(2 × mean negative control OD)] ×100. A sample E% <100% was classified as negative, and a sample E% ≥100% was classified as positive.

### *Post-mortem* Examination

Seventy-seven goats from the positive herd (herd A) were euthanized after ST reading by intravenous injection of a sodium pentobarbital overdose. A complete necropsy procedure was conducted for TB lesion examination. Lesions were collected and immediately fixed in 10% buffered formalin for histopathological confirmation by hematoxylin/eosin staining. Mediastinal and tracheobronchial lymph nodes (LNs) were removed and stored at −20°C for bacterial culture.

### Bacteriology

Whole pulmonary LNs of each animal were thawed, pooled, homogenized, and decontaminated as previously described (34) and plated on Middlebrook 7H11 medium (BD diagnostics, Sparks, MD, USA). Then, cultured plates were incubated at 37°C for 28 days. Finally, plates were read, and colonies were confirmed as MTBC by multiplex PCR (35).

### Data Analysis

The Sp was calculated in TB-free farms (herds B and C) using the formula Sp = True negatives/(True negatives + False positives). The Se was calculated in the TB-infected farm by the formula Se = True positive/(True positive + False negative). Clooper-Pearson 95% confidence intervals were calculated for Sp and Se. Differences in diagnostic results, between tests, were evaluated by the McNemar test. Moreover, agreement between tests was calculated by Cohen's Kappa coefficient (*k*) and interpreted as follows: <0.00 poor, 0.00–0.20 slight, 0.21–0.4 fair, 0.41–0.60 moderate, 0.61–0.80 substantial, and 0.81–1.00 almost perfect. The diagnostic performance of each test was calculated using the diagnostic odds ratio (DOR) (36). All statistical tests and 95% confidence intervals were calculated using the Epitools calculator (Sargento, ESG, 2018, Epitools Epidemiological Calculators, Ausvet, Pty., Ltd., Australia; available in www.epitools.ausvet.com.au).

## Results

The results of Se of herd A and Sp of herds B and C are summarized in Table 3. The TB-positive status of all animals from herd A was confirmed by positive mycobacterial culture and/or positive lesions in histopathological analysis.

**Table 3:** Sensitivity (Se) and specificity (Sp) of diagnostic tests.

**Diagnostic test**	**TB positive (farm A)**		**Unvaccinated (farm B)**		**BCG vaccinated (farm C)**	
	**N^9^**	**Se (95% CI)^10^**	**N**	**Sp (95% CI^11^**	**N**	**Sp (95% CI)^11^**
sP22-ST^1^	77	87% (77–94)	77	92% (84–97)	61	97% (89–100)
cP22-ST^2^	77	74% (63–83)	77	100% (95–100)	61	98% (91–100)
SIT^3^	77	94% (85–98)	77	94% (85–98)	61	67% (54–79)
SICCT^4^	77	91% (82–96)	77	100% (95–100)	61	80% (68–89)
sP22-IGRA^5^	77	91% (82–96)	74	95% (87–99)	68	84% (73–92)
cP22-IGRA^6^	77	86% (76–93)	74	96% (89–99)	68	85% (75–93)
STAND-IGRA^7^	77	77% (66–86)	74	100% (95–100)	68	96% (88–99)
DIVA-IGRA^8^	77	71% (60–81)	74	100% (95–100)	68	100% (95–100)
P22-ELISA	77	74% (63–83)	74	93% (85–98)	68	96% (88–99)
MPB83-ELISA	77	75% (64–84)	74	92% (83–97)	68	94% (86–98)

1 *sP22-ST, single P22 skin test*;

2 *cP22-ST, comparative P22 skin test*;

3 *SIT, single intradermal tuberculin test*;

4 *SICCT, single intradermal cervical comparative tuberculin test*;

5 *sP22-IGRA, single P22 IGRA test*;

6 *cP22-IGRA, comparative P22 IGRA test*;

7 *STAND-IGRA, standard tuberculin IGRA test*;

8 *DIVA-IGRA, differentiating Infected from Vaccinated animals (ESAT-6/CFP-10 peptide cocktail) IGRA test*.

9 *Number of animals tested*.

10 *Clopper–Pearson 95% confidence interval for Se*.

11 *Clopper–Pearson 95% confidence interval for Sp*.

*TB, tuberculosis; BCG, bacille Calmette-Guérin; IGRA, interferon-gamma release assay*.

### Skin Tests

The Se of the cP22-ST was the lowest among tests, but the Sp in herd B was the highest, being identical to the Sp of the SICCT, and a 6 percentage point (p.p.) and 8 p.p. more specific than the SIT and the sP22-ST, respectively. Regarding the herd C, the cP22-ST and the sP22-ST displayed similar Sp, being significantly more specific than the SIT (31 p.p. of increase, *p* < 0.001, and 30 p.p. of increase, *p* = 0.005, for cP22-ST and sP22-ST, respectively) and the SICCT (18 p.p. of increase, *p* = 0.0026, and 17 p.p. of increase, *p* = 0.0094, for cP22-ST and sP22-ST, respectively).

### Interferon-Gamma Release Assays

The sP22-IGRA showed the highest Se among tests, being a 5, 14, and 20 p.p. more sensitive than the cP22-IGRA, the STAND-IGRA, and the DIVA-IGRA, respectively. Indeed, the sP22-IGRA detected 12 positive goats more than the STAND-IGRA, without significant agreement between tests (*k* = 0.4, *p* = 0.098) and diagnostic results significantly different (Supplementary Data*, p* = 0.005). The sP22-IGRA and the cP22-IGRA showed similar specificities in both herds B and C, being a 4–5 p.p. less specific than the STAND-IGRA and the DIVA-IGRA. In herd C, both cP22-IGRA and sP22-IGRA were a 10–9 p.p. and a 16–15 p.p. less specific than the STAND-IGRA and the DIVA-IGRA, respectively.

### Serological Tests

In terms of Sp and Se, diagnostic results of P22-ELISA were similar to diagnostic results of MPB83-ELISA. In herd A, the MPB83-ELISA detected 10 TB positive animals more than the P22-ELISA, and the P22-ELISA detected nine TB positive animals more than the MPB83-ELISA, and the agreement between tests was considered fair although statistically significant (*k* = 0.35, *p* = 0.001). In herd B, diagnostic results of Sp showed a moderate but significant agreement between ELISA tests (*k* = 0.51, *p* < 0.001), but in herd C, no agreement was observed (*k* = −0.05, *p* = 0.33).

### Complementarity of Diagnostic Tests

Combined interpretation of P22-based tests was evaluated. Results of Sp and Se of complementarity of diagnostic tests are shown in Table 4. In general, complementarity between tests yielded an overall rise of Se with a variable reduction in the Sp.

**Table 4:** Sensitivity (Se) and specificity (Sp) combined results of P22-based diagnostic tests.

**Diagnostic tests**	**TB positive (farm A)**		**Unvaccinated (farm B)**		**BCG vaccinated (farm C)**	
	**N^8^**	**Se (95% CI)^9^**	**N**	**Sp (95% CI)^10^**	**N**	**Sp (95% CI)^10^**
SIT^1^ + sP22-IGRA^2^	77	97% (91–100)	73	89% (80–95)	61	59% (46–71)
SIT + cP22-IGRA^3^	77	97% (91–100)	73	90% (81–96)	61	61% (47–73)
SIT + P22-ELISA	77	96% (89–99)	73	89% (80–95)	61	66% (52–77)
SICCT^4^ + sP22-IGRA	77	97% (91–100)	73	95% (87–98)	61	67% (54–79)
SICCT + cP22-IGRA	77	97% (91–100)	73	96% (88–99)	61	67% (54–79)
SICCT + P22-ELISA	77	95% (87–99)	73	95% (87–98)	61	79% (66–88)
sP22-ST^5^ + sP22-IGRA	77	95% (87–99)	73	88% (78–94)	61	82% (70–91)
sP22-ST + P22-ELISA	77	94% (85–98)	73	88% (78–94)	61	93% (84–98)
cP22-ST^6^ + sP22-IGRA	77	95% (87–99)	73	95% (87–98)	61	84% (72–92)
cP22-ST + P22-ELISA	77	94% (85–98)	73	95% (87–98)	61	95% (86–99)
sP22-IGRA + STAND-IGRA^7^	77	92% (84–97)	74	95% (87–99)	68	84% (73–92)
P22-ELISA + sP22-IGRA	77	95% (87–99)	74	89% (80–95)	68	79% (68–88)
P22 ELISA + cP22-IGRA	77	95% (87–99)	74	91% (81–96)	68	81% (70–89)
P22-ELISA + MPB83-ELISA	77	87% (77–94)	74	92% (83–97)	68	90% (80–96)
P22-ELISA + STAND-IGRA	77	90% (81–95)	74	93% (85–98)	68	91% (82–97)
SIT + STAND-IGRA	77	95% (87–99)	73	93% (85–98)	61	67% (54–79)
SICCT + STAND-IGRA	77	95% (87–99)	73	100% (95–100)	61	80% (68–89)

1 *SIT, single intradermal tuberculin test*;

2 *sP22-IGRA, single P22 IGRA test*;

3 *cP22-IGRA, comparative P22 IGRA test*;

4 *SICCT, single intradermal cervical comparative intradermal tuberculin test*;

5 *sP22-ST, single P22 skin test*;

6 *cP22-ST, comparative P22 skin test*;

7 *STAND-IGRA, standard tuberculin IGRA test*.

8 *Number of animals tested*;

9 *Clopper–Pearson 95% confidence interval for Se*.

10 *Clopper–Pearson 95% confidence interval for Sp*.

*TB, tuberculosis; BCG, bacille Calmette-Guérin; IGRA, interferon-gamma release assay*.

The combination of cP22-ST + P22 ELISA improved the Se in 20 p.p. and displayed a similar Sp in both herds B and C, being the combined interpretation with the best results in all situations. The combination of SICCT + P22 ELISA showed similar results of Se and Sp in herd B. In herd C, the latter combination detected 10 false-positives more than the cP22-ST + P22-ELISA, reducing its Sp in 16 p.p., and with diagnostic results significantly different between tests (*p* = 0.004). The combination of cP22-ST + cP22-IGRA improved the Se and Sp in herd B at a similar level than the combined interpretations above described, but in herd C, the Sp was reduced in 11 p.p. respect to the cP22-ST + P22-ELISA test.

The combination of current diagnostic tests, e.g., SIT and SICCT, with other diagnostic tests increased the Se but not the Sp, except for the SICCT + STAND-IGRA. The latter combination improved the Se in 4 and 18 p.p. compared to the SICCT and the STAND-IGRA alone, respectively, and maintained the Sp in herd B but not in herd C (reduction of 16 p.p. compared to the STAND-IGRA alone). In herd A, the combined results of MPB83-ELISA + P22-ELISA improved the Se in 12 and 13 p.p. with respect to the MPB83-ELISA and the P22-ELISA alone, respectively, and maintained the Sp in herd B, and in herd C showed a mild reduction of Sp (4 and 6 p.p. of reduction with respect to the MPB83-ELISA and the P22-ELISA alone, respectively). Other combinations of tests did not improve the Se and the Sp, as did the aforementioned combined interpretations.

### Performance of Diagnostic Tests

**Figure 1:**
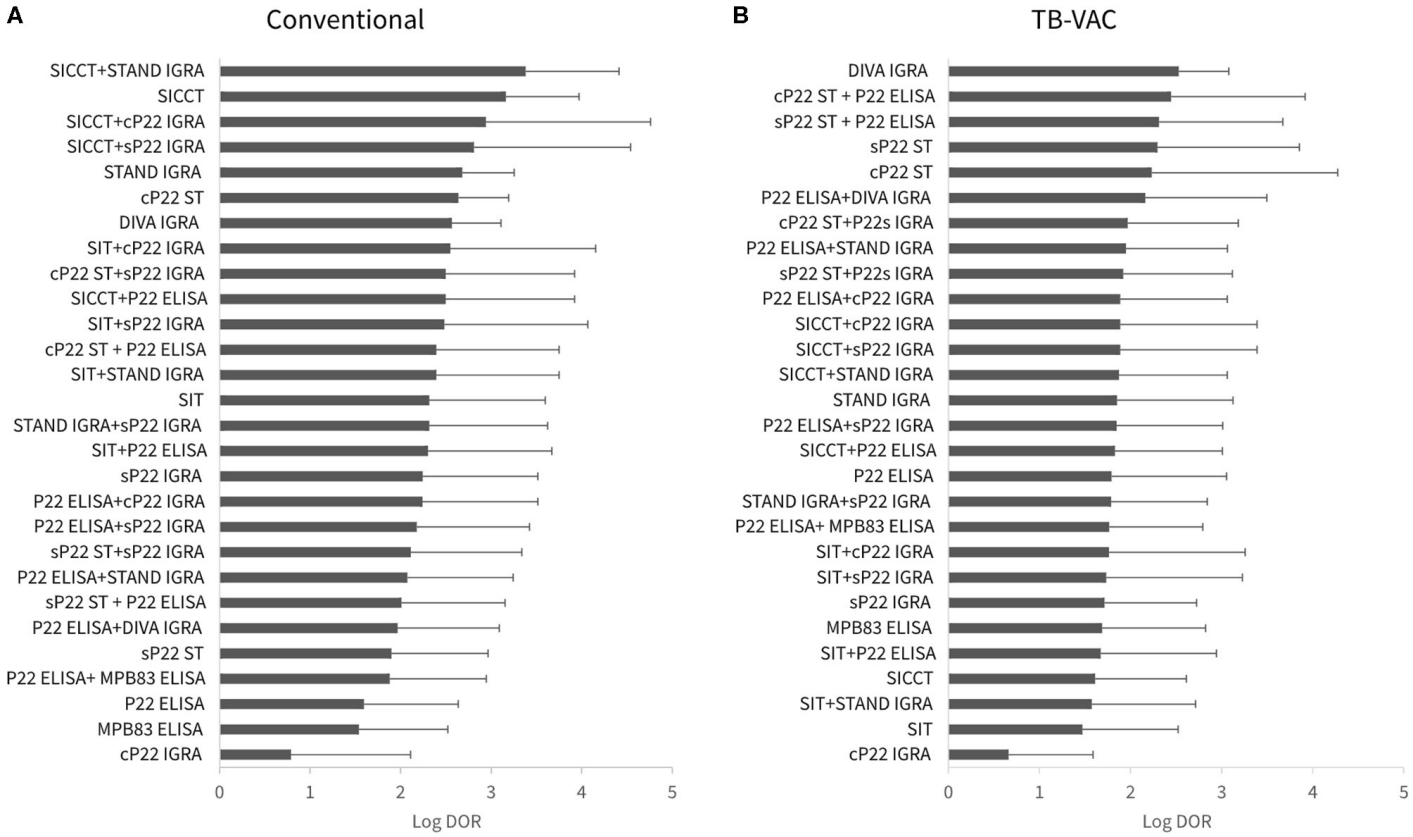
Diagnostic test performance measured by diagnostic odds ratio (DOR). **(A)** Conventional (unvaccinated scenario). **(B)** Tuberculosis (TB)-VAC scenario, animals were vaccinated with *Mycobacterium bovis* bacille Calmette-Guérin (BCG). sP22-ST, single P22 skin test; cP22-ST, comparative P22 intradermal skin test; SIT, single intradermal tuberculin test; SICCT, single intradermal cervical comparative tuberculin test; sP22-IGRA, single P22 interferon-gamma release assay (IGRA) test; cP22-IGRA, comparative P22 IGRA test; STAND-IGRA, standard tuberculin IGRA test; DIVA-IGRA, differentiating infected from vaccinated animals (ESAT-6/CFP-10 peptide cocktail) IGRA test.

The results of DOR to assess the diagnostic performance for each test are represented in Figure 1. In general, a reduced DOR in TB-VAC scenario was observed compared to the conventional one (0.47, 95% CI: 0.28–0.654, of mean reduction in log DOR). In the conventional context, SICCT + STAND-IGRA (3.38, 95% CI: 2.35–4.41), SICCT alone (3.16, 95% CI: 2.36–3.97), SICCT + cP22-IGRA (2.94, 95% CI: 1.12–4.76), and SICCT + sP22-IGRA (2.81, 95% CI: 1.08–4.54) showed the best performances (Figure 1A). In TB-VAC context, the best performances were observed in DIVA IGRA (2.53, 95% CI: 1.98–3.8), cP22-ST + P22 ELISA (2.44, 95% CI: 0.97–3.92), and sP22-ST + P22 ELISA (2.31, 95% CI: 0.95–3.67) (Figure 1B).

## Discussion

Efficient and accurate diagnosis is of paramount importance for the success of eradication programs based on test and slaughter strategy. Here, the performance of new P22 antigenic complex-based cell-mediated and humoral tests for the diagnosis of TB in goats was assessed under different epidemiological and TB control scenarios.

Recently, the P22 antigenic complex has been evaluated for the detection of IgG in ELISA tests in different species: cattle goat, sheep, pigs, and wild boar (24–27), red deer (28), badgers (29), and alpacas and llamas (30). In the present study, the performance of the P22 antigenic complex for diagnostic tests based on CMI, namely, STs and IGRA, was evaluated for the first time in goats. Indeed, the use of P22 for IGRA tests has only been reported in red deer experimentally infected with *M. bovis* (37).

The combined interpretation of tests leads to a substantial improvement of Se at the expense of a variable loss of Sp. As expected, in the conventional context, the SICCT alone or combined with the STAND-IGRA (8, 11, 38) showed the best performances by DOR analysis. The performances of tuberculin-based tests were followed by the combinations of SICCT with the P22-IGRAs, which increased Se at the cost of a certain loss in Sp. Moreover, the combination of cP22-ST + P22-ELISA clearly increased the Se with the benefit of a minimal decrease of Sp, showing similar results than the combination of SICCT + P22-ELISA. These findings are in concordance with previous studies of P22-ELISA and tuberculin-based skin testing. In cattle, the combination of SIT + P22-ELISA showed an improvement of Se of 30 and 6 p.p. compared to the SIT and the P22-ELISA alone, respectively (25). In another study conducted in goats (39), the same combination improved the Se of the SIT and the P22-ELISA in 19 and 9.5 p.p., respectively. Also, in the same study in goats, the combination of SICCT + P22-ELISA improved the Se of the SICCT in a 24 p.p. These results confirmed the benefits of the strategic use of serological and CMI-based diagnostic tests in parallel to maximize the Se in infected settings.

In the TB-VAC context, the combination of P22-ELISA with the two P22-based STs showed similar performances than the DIVA-IGRA. However, the latter showed considerably lower Se than the combinations of P22-ELISA with P22-based STs (reduction in 23–24 p.p.). Previous studies reported the excellent Sp (16) and the lack of Se (40) of DIVA-IGRA, although the DOR analysis tended to overestimate the Sp in this study. The Se of vaccine-associated diagnostic tests is an essential requirement for the development of an integral vaccination strategy (41), and the combination of cP22-ST + P22-ELISA showed an efficient and innovative diagnostic approach in the TB-VAC context, showing the highest combined Se and Sp values (94 and 95%, respectively).

Concerning the use of the ST in solitary, the P22-based STs showed lower Se compared to both the SIT and the SICCT tests, although previous studies in dairy goat flocks, with larger samples and different epidemiological situations, have shown lower Se for SIT (65%, 95% CI: 63.3–68.2) (8) and SICCT (44.5%, 95% CI: 35–55) (42). However, the Se of the cP22-ST (74%, 95% CI: 63–83) was similar to Se observed in two previous studies using DIVA STs (based on the peptide cocktails ESAT-6, CFP-10, and Rv3616c) developed for the diagnosis of TB in cattle: 76%, 95% CI: 59–93 (43) and 75%, 95% CI: 47.7–97.7 (44). In the latter, the addition of the Rv3020c peptide improved the Se to reach 87.5% (95% CI: 61.7–98.5), being similar to the Se of sP22-ST (87%, 95% CI: 74–94) obtained in the present study. On the other hand, in BCG-vaccinated animals, the Sp of SIT and SICCT decreased dramatically (27 and 20 p.p. of reduction, respectively), whereas the Sp of sP22-ST and cP22-ST remained high (97 and 98%, respectively). These findings again highlight the suitability of P22-based STs as TB vaccine-associated diagnostic candidates, although improvements to increase the Se should be necessary.

Moreover, herd PTB status and MAP vaccination may also affect the interpretation of the results. MAP infection was not reported in farms B and C, and no recent clinical history of PTB was observed by the veterinarians. Despite this, vaccination against MAP is a common practice in small ruminants in Spain (12), and diagnostic interferences due to MAP vaccination on TB diagnosis cannot be ruled out in these two MAP-vaccinated herds. In this sense, strong reactions to PPD-A were observed at skin testing (Supplementary Data), but the results of comparative tests (cP22-ST and SICCT) showed higher Sp compared to their respective single STs (i.e., sP22-ST and SIT). These findings indicate that some degree of cross-reactivity due to MAP vaccination was still maintained. Similarly, interferences of MAP vaccination on TB diagnosis, mainly in CMI-based diagnostic tests, were previously observed in MAP-vaccinated goats (14, 45).

Surprisingly, the P22-based IGRAs, particularly the sP22-IGRA, showed higher Se compared to STAND-IGRA and even higher compared to DIVA-IGRA. However, the Se of sP22-IGRA was similar to that previously observed by the STAND-IGRA (92%, 95% CI: 84–96) in other studies conducted in goats (26). The results of Se of the cP22-IGRA in the present study were also similar to those previously observed in experimentally *M. bovis*-infected red deer (37). However, a slight loss of Sp in the P22-IGRAs was detected compared to STAND-IGRA. Even so, the Sp was within ranges (95–100%) described for STAND-IGRA in previous studies (11, 38, 45). This mild reduction in Sp could be explained by the high concentration of P22 used for stimulation of whole blood (20 μg/ml) and by the fact that P22 complex contains 21 proteins also present in *M. avium* (23), which can cause cross-reactivity with MAP vaccination and/or infection. Indeed, the interference of MAP vaccination on STAND-IGRA has been previously observed in adult MAP-vaccinated goats (13, 14, 45). The Sp of P22-IGRAs considerably decreased in BCG-vaccinated herds compared to that previously described for the STAND-IGRA (16). Overall, the results of sP22-IGRA suggest that this test could be a potentially valuable tool for TB eradication in endemic areas, although further studies to determine the optimal concentration of P22 are required to improve its Sp with a minimal loss of Se.

Serological diagnostics is a cost-effective alternative for TB diagnostics. However, the Se of antibody-based diagnostic tests was generally lower compared to tests based on CMI (46, 47). In the present study, the Se of P22-ELISA was slightly lower than that in previous studies in goats and cattle (25, 39). This loss of Sp might be explained by the fact that animals from herd A were not vaccinated against MAP nor subjected to frequent intradermal testing, factors that could enhance humoral responses against MTBC antigens (48). Interestingly, the Se was significantly enhanced when using P22 and MPB83 ELISAs in parallel. Thus, even though MPB83 is a major component of the P22 complex, specific IgGs of some infected animals were only detectable using the MPB83 purified recombinant protein alone, while others were only detected using the P22 complex, which contains additional serodominant epitopes (23).

Finally, Sp of the P22-ELISA reached considerably higher Sp in MAP-vaccinated (and BCG-unvaccinated, i.e., herd B) goats (93%) compared to that previously found in Spanish (78%) and Norwegian MAP-vaccinated goats (58%) (24). In the latter study, besides MAP vaccination, MAP coinfection and/or contact with environmental mycobacteria was not discarded as a source of diagnostic interference. Interestingly, in the present study, the Sp was also high in BCG- and MAP-vaccinated goats (96%), suggesting that BCG vaccination does not induce antibody responses that cause interference on the diagnosis by the P22-ELISA. The absence of antibody responses was consistent with the fact that the BCG Danish strain used for vaccination expresses low levels of MPB83 and MPB70 (49), which are the most abundant proteins of the P22 antigenic complex (23). Moreover, tuberculin skin testing after 42 days of MAP or BCG vaccination caused a boosting effect on humoral responses against tuberculin antigens, resulting in false-positive cattle for an MPB83-based ELISA (50). Here, minimal or no boosting effects of MAP/BCG vaccination due to skin testing were observed on the P22-ELISA. Indeed, goats from herd B were sampled around 2 years after vaccination against MAP, and ST was performed once or twice after MAP vaccination. Also, 34/68 goats from herd C were vaccinated with BCG and Gudair^®^ at 9–10 months before the sampling, whereas the rest of the animals were vaccinated more than 1 year before, and no ST was performed since. Based on the results herein, the P22-ELISA seemed to be a useful ancillary diagnostic tool, either in BCG or MAP vaccination context, although it should be confirmed in further studies with larger sized herds.

In conclusion, this study reinforces the applicability of the P22 antigen complex as a complementary instrument for TB diagnostics in goats under different control scenarios. The P22 serological diagnostic is a cost-effective alternative, and combined interpretation with STs, either with PPD-B or P22, showed promising results. Moreover, the use of P22 antigenic complex in CMI-based diagnostic tests showed encouraging results, being suitable for further research on the improvement of TB diagnostics.

## Data Availability Statement

All datasets generated for this study are included in the Supplementary Material.

## Ethics Statement

All animals included in this study belonged to commercial farms and were not experimental animals. All sampling and handling procedures were carried out by authorized veterinarians according to standard farm methods and in conformity with Spanish legislation (Royal Decree 2611/1996 and amendments) and European Union laws for the protection of animals used for scientific purposes (2010/63/EU). Test and slaughter of positive animals, as well as *post-mortem* sampling to confirm the disease, were conducted according to the regulations defined by the Catalan Government (Resolution AAM/1314/2014). Written informed consent was obtained from the owners for the participation of their animals in this study.

## Author Contributions

BP and JB contributed to conceptualization. CA-V, BP, and MG contributed to data curation. CA-V and BP performed the formal analysis. BP and LJ acquired funding. CA-V, BP, MG, EV, MD, IM, JI-L, and JB contributed to the investigation. CA-V, BP, JI-L, EV, and MD contributed to the methodology. BP and LJ contributed to project administration. JI-L and MS acquired resources. CA-V and BP wrote the original draft. JB, LJ, JI-L, EV, and MD contributed to writing, reviewing, and editing.

## Conflict of Interest

The authors declare that the research was conducted in the absence of any commercial or financial relationships that could be construed as a potential conflict of interest.

## Supplementary Material

Supplementary Data Raw data diagnostic tests.
